# Artificial Food for Children

**Published:** 1884-06-20

**Authors:** 


**Artificial Food for Children.**—There has been great discussion as to the qualifications of condensed milk as a substitute for the human article. Some men strongly advocate its use, while others bitterly condemn it. After reporting a case of infantile scurvy, in a foreign exchange, Dr. Edmond Owen says :

"The opinion which I have been compelled to form in my work in the out-patient rooms of the Children's Hospital, is that the worst nourished of the hand-fed infants are those that have been reared upon condensed milk and the various patent food stuffs; and that whenever an infant cannot have human breast-milk, the best substitute will be found in fresh cow's milk prepared and administered *secundum artem.*—*Med. and Surg. Rep.*, March 29, 1884. [The above must be taken with some degree of allowance. We have in a number of instances seen good results follow the use of condensed milk in cases where the cow's milk was not tolerated.—ED. REC.]

